# ExoDx prostate test as a predictor of outcomes of high-grade prostate cancer – an interim analysis

**DOI:** 10.1038/s41391-023-00675-1

**Published:** 2023-05-16

**Authors:** Ronald Tutrone, Ben Lowentritt, Brian Neuman, Michael J. Donovan, Elliot Hallmark, T. Jeffrey Cole, Yiyuan Yao, Claire Biesecker, Sonia Kumar, Vinita Verma, Grannum R. Sant, Jason Alter, Johan Skog

**Affiliations:** 1 Chesapeake Urology Research Associates, Baltimore, MD USA; 2 Icahn School of Medicine at Mt. Sinai, New York City, NY USA; 3 Exosome Diagnostics, a Bio-Techne Brand, Waltham, MA USA; 4 Department of Urology, Tufts University, Medford, MA USA

**Keywords:** Cancer, Outcomes research

## Abstract

**Background:**

Patient outcomes were assessed based on a pre-biopsy ExoDx Prostate (EPI) score at 2.5 years of the 5-year follow-up of ongoing prostate biopsy Decision Impact Trial of the ExoDx Prostate (IntelliScore).

**Methods:**

Prospective, blinded, randomized, multisite clinical utility study was conducted from June 2017 to May 2018 (NCT03235687). Urine samples were collected from 1049 men (≥50 years old) with a PSA 2–10 ng/mL being considered for a prostate biopsy. Patients were randomized to EPI vs. standard of care (SOC). All had an EPI test, but only EPI arm received results during biopsy decision process. Clinical outcomes, time to biopsy and pathology were assessed among low (<15.6) or high (≥15.6) EPI scores.

**Results:**

At 2.5 years, 833 patients had follow-up data. In the EPI arm, biopsy rates remained lower for low-risk EPI scores than high-risk EPI scores (44.6% vs 79.0%, *p* < 0.001), whereas biopsy rates were identical in SOC arm regardless of EPI score (59.6% vs 58.8%, *p* = 0.99). Also in the EPI arm, the average time from EPI testing to first biopsy was longer for low-risk EPI scores compared to high-risk EPI scores (216 vs. 69 days; *p* < 0.001). Similarly, the time to first biopsy was longer with EPI low-risk scores in EPI arm compared to EPI low-risk scores in SOC arm (216 vs 80 days; *p* < 0.001). At 2.5 years, patients with low-risk EPI scores from both arms had less HGPC than high-risk EPI score patients (7.9% vs 26.8%, *p* < 0.001) and the EPI arm found 21.8% more HGPC than the SOC arm.

**Conclusions:**

This follow-up analysis captures subsequent biopsy outcomes and demonstrates that men receiving EPI low-risk scores (<15.6) significantly defer the time to first biopsy and remain at a very low pathologic risk by 2.5-years after the initial study. The EPI test risk stratification identified low-risk patients that were not found with the SOC.

## Introduction

PSA screening allows for early detection of tumors but is very non-specific and elevated PSAs are often the result of benign prostatic hyperplasia (BPH). Additionally, PSA testing enables a higher detection rate of indolent tumors, as the PSA test is not able to discriminate high-grade PC (HGPC) [1, 2]. Due to the non-specific nature of PSA testing, screening recommendations have ranged from avoiding screening altogether, to promoting age-specific and decision-based PSA testing for patients aged 55–69 [3].

The ExoDx Prostate, EPI test, is a urine exosome gene expression assay that does not require a pre-collection digital rectal exam (DRE) to make informed prostate biopsy decisions. The EPI test performance has been extensively studied previously [4–8] and has a negative predictive value (NPV) of 91% for ≥GG2 and 97% for ≥GG3 [8]. EPI does not include any clinical/standard-of-care features, and is a standalone test that provides a risk stratification/assessment score to discriminate between no cancer/low-grade PC (Gleason Grade Group 1 [GG1], EPI ≤ 15.6) and HGPC (≥GG2, EPI > 15.6) [8]. The EPI test has been included in the National Comprehensive Cancer Network guidelines for Prostate Cancer Early Detection since 2019 [9]. Previously, we partnered with a major healthcare insurer to execute a unique, prospective, randomized, blinded, two-armed clinical utility study to determine the impact of the EPI test on the biopsy decision-making process between patients and urologists [4]. The utility study found that in the blinded control arm, many high-risk patients still opted out of doing the recommended biopsy, and doctors that had access to the EPI result found 30% more HGPC compared to the blinded arm using standard of care methods. Patients with a low-risk score deferred their biopsies and the control arm showed that these low-risk men that can defer a biopsy were not identified with SOC. Here, we present a retrospective qualitative electronic medical record (EMR) chart review of the results for patients at 2.5 years of the 5-year follow-up plan. We compare subsequent biopsies and associated pathology in EPI high- vs EPI low-risk men over time as well as how EPI results impact time to biopsy.

## Materials and methods

### Study population

Briefly, a prospective, blinded, randomized, multicenter clinical utility study from June 2017 to May 2018 (Decision Impact Trial of the ExoDx Prostate (IntelliScore), NCT03235687) evaluated the shared decision impact of the EPI test. This study was approved by the Institutional Review Board at Western IRB (#20171107) and conforms to the principles outlined in the Declaration of Helsinki. Inclusion criteria required that patients be men aged ≥50, with PSA 2–10 ng/mL, and without clinical history of prior biopsy scheduled for an initial prostate biopsy.^7^ Patients with a recent (<6 months) history of invasive treatment for benign prostatic disease or taking medications affecting PSA levels within 6 months were excluded. All eligible participants provided written and informed consent. Once enrolled, patients provided a urine sample and had an EPI test, and were subsequently randomized into two groups: those who would receive the EPI result as part of their biopsy decision process (EPI), and those who would not, and would instead receive “standard of care” (SOC) treatment including PSA, patient age, family history, DRE results, PSA density, % free PSA, nomograms, mpMRI (22%) and potentially other commercially available biomarker tests. In this study, patients returned to routine standard of care after enrollment in the clinical utility trial, and a retrospective outcome analysis was conducted 2.5 years after study initiation as part of a planned five-year follow up. Follow-up parameters including biopsy pathology outcomes were analyzed from the single baseline EPI result obtained in the original clinical utility trial.

### Statistical analysis

Statistical differences in clinical and demographic categorical variables (e.g. race) impacting on the biopsy decision process were estimated with a Pearson’s chi-squared test. Continuous variables (e.g. age, PSA) were tested for normality using the Shapiro–Wilk test. Parametric distributions of two variables were compared using a Welch’s two-sample *t*-test, and comparison of more than two normally distributed variables were compared using one-way ANOVA. Non-parametric distributions of more than two variables were compared using a Kruskal–Wallis rank-sum test, followed by Dunn’s test for multiple pairwise comparison using Benjamini-Hochberg adjustment for the *p*-value. All comparisons of counts between groups were assessed using Pearson’s Chi-squared goodness of fit test. The *p*-value threshold for significance for all statistical tests was set to *p* < 0.05. All statistical analyses and plots were generated using R version 4.0.3.

## Results

**Fig. 1:**
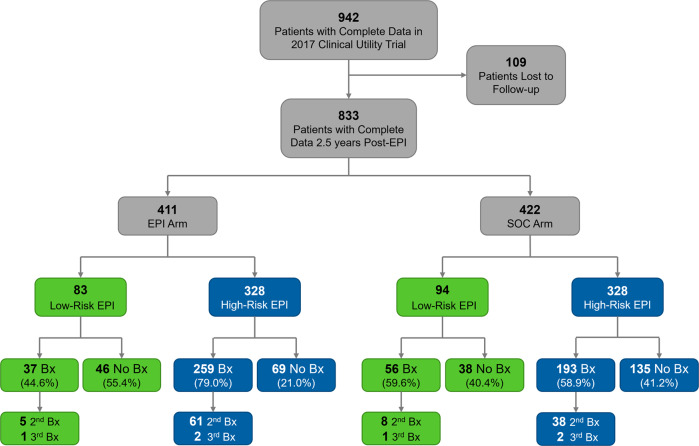
Consort Flow Diagram. A total of 72 urologists from 24 clinical sites enrolled patients 942 for the study, of which 109 were lost to follow-up. Only the EPI arm received the initial EPI result back. Patients in the SOC arm did not receive the EPI result back, and instead relied on standard of care for all biopsy decisions at the time of enrollment, and throughout the 2.5-year follow up. Bx Biopsy, EPI ExoDx Prostate (IntelliScore), SOC Standard of Care.

Of the original cohort of 1094 patients in the clinical utility trial, complete data and usable samples were available for 942 patients, and 833 of those patients had complete follow-up data at 2.5 years (Fig. 1, Table 1). The patient demographics were 68.7% Caucasian, 22.3% African American, 2.8% Asian and 1.8% Hispanic. Most patients had a non-suspicious DRE (86.7%), and no family history of PC (84.5%). As defined by the inclusion criteria, patients in each arm had equivocal PSA values with a median of 5.04 (range 2.0–10.0, Table 1). Results were analyzed in two ways: (1) Clinical pathology outcomes were assessed in the two arms, where the randomized control arm did not receive the EPI result back and had to rely only on standard of care; and (2) Biopsy deferrals, as well as time to biopsy were compared between study arms for cases with low- or high-risk EPI scores.

**Table 1:** Patient Demographics and Disease Characteristic.

	EPI Arm (*n* = 411)	SOC Arm (*n* = 422)	Statistics
Age, median (IQR)	65 (59–69)	65 (59–70)	W = 83583.5, *p* = 0.37
PSA, median (IQR)	4.8 (4.0–6.0)	4.8 (3.6–6.1)	W = 89058.5, *p* = 0.5
EPI Test Score, median (IQR)	29.6 (17.7–46.8)	28.2 (16.6–42.7)	W = 90935.5, *p* = 0.22
PC Family History, *n* (%)			χ^2^ = 0.20, *p* = 0.90
Yes	61 (14.8)	59 (14.0)	
No	346 (84.2)	358 (84.8)	
Unknown	4 (1.0)	5 (1.2)	
Ethnicity, *n* (%)			χ^2^ = 4.24, *p* = 0.52
Black	89 (21.7)	97 (23.0)	
Asian / Pacific Islander	13 (3.2)	10 (2.4)	
White	287 (69.8)	285 (67.5)	
Hispanic	7 (1.7)	8 (1.9)	
Other	8 (1.9)	17 (4.0)	
Unknown	7 (1.7)	5 (1.2)	
DRE, *n* (%)			χ^2^ = 4.73, *p* = 0.09
Non-suspicious	365 (88.8)	357 (84.6)	
Suspicious	21 (5.1)	22 (5.2)	
Unavailable	25 (6.1)	43 (10.2)	

*DRE* digital rectal exam, *EPI* ExoDx Prostate (IntelliScore), *PC* prostate cancer, *PSA* prostate specific antigen

### 2.5-year clinical outcomes

#### Biopsy Rates

Both study arms, EPI arm (*N* = 411) and the SOC arm (*N* = 422) included a total of 833 patients, of which 177 had low-risk EPI scores and 656 had high-risk EPI scores. Out of all patients, 34.6% (*N* = 288/833) did not have a prostate biopsy (Table 2, Fig. 1), 51.3% (*N* = 427/833) had pathology data from a single biopsy, 13.4% (*N* = 112/833) had two biopsies, and 0.7% (*N* = 6/833) had three biopsies over the course of the 2.5-year follow-up. The MRI utilization, 22% of men (*N* = 183/833), reflected the MRI use within Chesapeake Urology during the study period [4].

**Table 2:** EPI Scores and Gleason Grades.

*n* (%)	Arms Combined		EPI Arm		SOC Arm	
	EPI ≥ 15.6	EPI < 15.6	EPI ≥ 15.6	EPI < 15.6	EPI ≥ 15.6	EPI < 15.6
	*n* = 656, (%)	*n* = 177, (%)	*n* = 328, (%)	*n* = 83, (%)	*n* = 328, (%)	*n* = 94, (%)
**Deferred**	204 (31.1)	84 (47.5)	69 (21.0)	46 (55.4)	135 (41.2)	38 (40.4)
**Benign**	179 (27.3)	69 (39.0)	98 (29.9)	30 (36.1)	81 (24.7)	39 (41.4)
**GG 1**	94 (14.3)	10 (5.6)	59 (18.0)	3 (3.6)	35 (10.7)	7 (7.4)
**GG 2**	90 (13.7)	8 (4.5)	54 (16.5)	3 (3.6)	36 (11.0)	5 (5.3)
**GG 3**	56 (8.5)	5 (2.8)	30 (9.1)	1 (1.2)	26 (7.9)	4 (4.3)
**GG 4**	15 (2.3)	0 (0.0)	9 (2.7)	0 (0.0)	6 (1.8)	0 (0.0)
**GG 5**	18 (2.7)	1 (0.6)	9 (2.7)	0 (0.0)	9 (2.7)	1 (1.1)
**GG** ≥ **2**	179 (27.3)	14 (7.9)	102 (31.1)	4 (4.8)	77 (23.5)	10 (10.6)

*EPI* ExoDx Prostate (IntelliScore), *GG* Gleason Grade, *SOC* Standard of Care

**Fig. 2:**
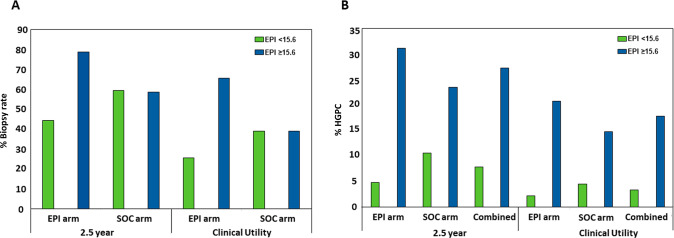
Patient Biopsy Rates and GG ≥ 2 HGPC Probability by EPI Score. Comparison of biopsy rate and HGPC (≥GG2) in the randomized EPI vs control (SOC) arm at the time points of the clinical utility trial vs after a 2.5 year follow-up. **A** In both the original Clinical Utility study and the 2.5-year follow-up data, patients with low-risk (<15.6) EPI scores in the EPI arm had a significantly lower biopsy rate than patients with high-risk EPI scores, while patients in the SOC arm deferred biopsies at almost identical rates in the low vs high risk EPI patients. **B** After 2.5 years follow-up, patients with low-risk EPI scores had very low probability of a HGPC (≥GG2) diagnosis, while patients with high-risk EPI scores had much higher probability of HGPC diagnosis, regardless of study arm. This was consistent in the original Clinical Utility data and in the 2.5 year follow-up data. The percentage of HGPC cancer found includes the total patient number in the arm, including patients that did not receive a biopsy.

The average time from EPI testing to the first biopsy was significantly longer in the low-risk EPI arm (216 days), compared to EPI high-risk (68.7 days; *p* < 0.001) and when compared to the low-risk EPI patients in the SOC arm (79.4 days; *p* < 0.001). These low-risk patients could not be identified by standard of care and they received a biopsy despite their lower HGPC risk, whereas they were identified and deferred in patients that received the EPI score. Conclusions on time to second or third biopsies could not be drawn due to few events. When comparing EPI results by study arm, we observed that in the EPI arm, low-risk patients were much less likely to have biopsies than high-risk patients (44.6% vs 79.0%, *p* < 0.001; Fig. 2A), whereas in the SOC arm, the choice to defer was independent of EPI and therefore, not different between low-risk and high-risk EPI scores (59.6% vs 58.8%, *p* = 0.99; Fig. 2A). While patients had more biopsies over time, this trend was similar to that observed in the utility study with fewer biopsies in the EPI arm for low-risk patients (25.8% vs 65.8%, *p* < 0.001; Fig. 2A), whereas the rates of low- and high-risk EPI were similar in the SOC arm (39.3% vs 39.2%, *p* = 0.994; Fig. 2A). The reduction of biopsies in patients with low-risk EPI scores in the EPI arm compared to the SOC arm is also notable (44.6% vs 59.6%, *p* < 0.04617). The identical biopsy rates in the SOC arm among the low-risk EPI and high-risk EPI results highlights that EPI provides independent information allowing low-risk patients to be identified which was not possible by SOC factors.

#### Biopsy outcomes

**Fig. 3:**
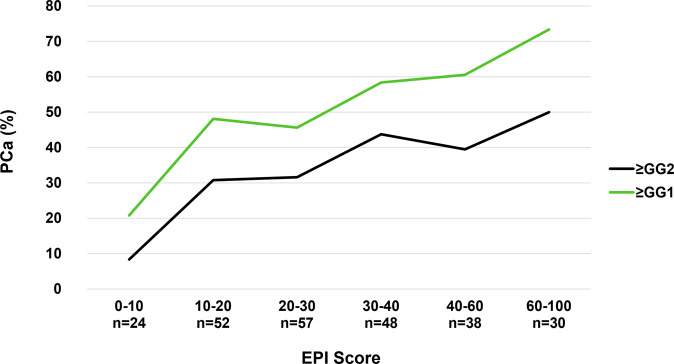
Overall Probability of Identifying ≥ GG2 PC by EPI Score in the SOC Arm. The SOC arm was used to plot the risk of finding HGPC as a function of the EPI score (the EPI arm utilized the score in the decision to biopsy and cannot be used for performance). This graph overlaps well with the increasing risk scores from previous validation studies.^8^ Increasing EPI score correlates with increasing likelihood of finding HGPC. The likelihood of finding HGPC is limited by the 12-core TRUS biopsy, which has a sensitivity of approximately 48–50% for finding HGPC, therefore, the probability of finding cancer beyond 50% will be limited. EPI ExoDx Prostate, GG Gleason Grade, HGPC High-grade prostate cancer.

The EPI arm received a single EPI test prior to the biopsy decision and the SOC arm received standard of care that could include other testing. At the end of the utility study, the EPI arm had found 30% more HGPC than the control arm. The low-risk patients that could defer a biopsy were not identified with the SOC parameters, as evaluated by the blinded EPI scores in the SOC arm (equal biopsy rate among high- and low-risk EPI in the blinded control arm). There was initially a high deferral rate of the biopsy in the SOC arm (60.7%), but it was equally distributed across EPI high- and low-risk patients and led to missing more HGPC in the SOC arm compared to the EPI arm. The additional biopsies performed over the next 2.5 years found additional cancer in both arms, but the EPI arm still found 21.8% more HGPC (≥GG2) than in the SOC arm (106 vs 87 HGPC). The biopsy deferral rate decreased in the SOC arm from 60.3% to 41%, and the deferral was again equally distributed across the high and low EPI scores (blinded to the urologist), indicating that EPI provides a unique value and SOC could not stratify the low- vs high-risk patients. In contrast, the biopsy deferral in the EPI arm 2.5 years later was still significantly higher in the low-risk EPI group vs high-risk group (55.4% vs 21%) (Supplemental Table 1). Furthermore, the % of biopsies that found HGPC (≥GG2) significantly decreased in the SOC arm from 31.6% at initial biopsy to 20.6% during the follow-up. The ratio of biopsies finding HGPC in the EPI arm was only reduced to 25.8% during the follow up. Overall, patients with high-risk EPI scores were approximately six times more likely to have HGPC after 2.5 years (4.8% vs 31.1%, *p* < 0.05) if you received the EPI result and approximately twice as likely if you were blinded to the EPI result and could not act on it (10.6% vs 23.5%, *p* < 0.05, Fig. 2B). Of note, only 3.4% (6/177) of EPI low-risk results had ≥GG3 of which 1 patient (0.6%) had >GG3 after 2.5 years of follow-up. The performance of the EPI test in the SOC cohorts aligns with the correlation noted in a previous publication [6] which demonstrates a linear relationship between the percent likelihood of having HGPC and an increasing EPI score (Fig. 3).

## Discussion

Evidence from a previously reported prospective randomized, clinical utility study established that the ExoDx Prostate (EPI) test significantly impacts prostate biopsy decisions [4]. In the initial clinical utility study, we observed that the EPI arm resulted in 23% more biopsies than the SOC arm, but biopsies were more appropriately applied: this resulted in the EPI arm detecting 30% more HGPCA (≥GG2) than the SOC arm, and low-risk men deferred their biopsies. We now report on the follow-up data at 2.5 years post-trial enrollment, by assessing patient outcomes over time through analysis of subsequent changes in biopsy decisions and timing as well as the resultant pathology. When EPI results were evaluated by study arm, no difference in biopsy deferral was observed in the SOC arm. In the EPI arm, EPI low-risk patients deferred prostate biopsies at a significantly higher rate than patients who had EPI high-risk results. This shows that SOC fail to properly identify the low- vs high-risk patients and the biopsy deferral seemed random across the EPI risk scores in the SOC arm. Moreover, the time from EPI testing to first biopsy was significantly longer when EPI results were low-risk compared to high-risk (216 vs. 68.7 days respectively).

Patients from the EPI and SOC arms were combined and split into two groups based on the initial EPI risk assessment of low- or high-risk for HGPC ( ≥ GG2), using the previously established risk threshold of 15.6 [7, 8]. We observed that EPI low-risk patients ( < 15.6) followed for 2.5 years from either arm had a low rate (7.9%, 14/177) of high-grade prostate cancer ( ≥ GG2) detected upon biopsy. Furthermore, only 3.4% (6/177) of EPI low-risk results had ≥GG3 of which 1 patient (0.6%) had >GG3 after 2.5 years of follow-up. These outcomes are similar to the prior validation studies demonstrating low incidence of clinically significant high-risk PC in EPI low-risk cases at the time of biopsy [7, 8]. Conversely, patients with EPI high-risk scores (≥15.6) had a higher incidence of high-grade disease: ≥GG2 disease (27.3% (179/656)), GG3 (8.5% (56/656)) and >GG3 (5.0% (33/656)). In oncology, survival or metastasis are important key outcome measures, but these metrics are difficult to assess in slow-growing diseases like prostate cancer. In fact, only a few long-term, randomized, controlled prostate cancer mortality trials have been completed [10–12].

A key context to understanding EPI low-risk results is the fact that nearly all patients with subsequent diagnosis of GG1 disease will have favorable long-term mortality outcomes [13–15]. Early modeling studies suggested that patients with <GG2 cancer had <2% risk of mortality at 15 years [16]. Indeed, active surveillance (AS) became an established tool for patients with favorable risk PC (GG1) because AS can reduce overtreatment without negative clinical impact [17–19]. Patients with low-risk disease managed with AS have excellent cancer-specific (99.9%) and metastasis-free (99.4%) survival at 10 and 15 years (99.9% and 99.4% respectively) [20]. In this study, 4.5% of EPI low-risk results, were observed to have GG2 which, depending upon volume, can be defined as favorable intermediate-risk disease. AS can be used for favorable intermediate-risk prostate cancer when clinical features meet specific criteria [21, 22].

During the study period, there was limited use of mpMRI (22%) and no use of PSMA imaging in this cohort. Thus, this reflects the reality of implementing a clinical follow-up research program as medical practice evolves. In current practice, mpMRI is more prevalent and accepted in early prostate cancer detection, but PSMA imaging is not used pre-diagnosis. Moreover, F-18 piflufolastat and Ga-68 PSMA-11 are indicated for post-treatment biochemical recurrence or unfavorable intermediate- or high-risk localized disease [23]. Biomarkers are still very relevant within the context of expensive imaging modalities, which like all risk assessment methods, have limitations and areas for improvement [23–26]. Current discussions now focus on how biomarkers and mpMRI should work together as they appear complementary [23, 27]. Biomarker assays, such as ExoDx Prostate, provide independent risk information and may be layered together to provide a more informed risk assessment [27–33]. ExoDx Prostate has demonstrated complementary value to mpMRI and is used in contemporary practice with mpMRI [28, 34]. Developing risk assessment tools that can combine the benefits of mpMRI with biomarkers is underway [35] and an important area of future study.

### Limitations

The current study has limitations, mostly related to the overall small sample size of men proceeding with additional biopsies post enrollment, potential selection bias, and limited use of mpMRI (22%). However, this study represents real world evidence of clinical practice during the initial study and subsequent 2.5-year follow-up period.

## Conclusion

This unique follow up study highlights the outcome of a pre-biopsy EPI score and how it changes the clinical outcome for patients even 2.5 years later compared to a blinded control arm that could utilize any standard of care parameters, including other biomarkers. The prior EPI clinical utility study trial demonstrated that EPI significantly impacts prostate biopsy shared decision-making during the decision process. This study extends the initial analysis by demonstrating that EPI continues to contribute risk information over time that is not available with SOC parameters. Men with EPI low-risk scores safely defer testing for longer and, based on biopsy pathology, remain low-risk for years. Only 1.2% of patients in the EPI arm and 3.4% in the combined cohort defined as EPI low-risk had ≥GG3 prostate cancer across multiple biopsies at 2.5 years. Overall, this study demonstrates that a pre-biopsy EPI test leads to better outcomes even when followed for 2.5 years after the initial biopsy decision-making process.

## Supplementary information

Supplemental Material

## Data Availability

The datasets used and/or analysed during the current study are available from the corresponding author on reasonable request.
