# Correction to: ExoDx prostate test as a predictor of outcomes of high-grade prostate cancer – an interim analysis

**DOI:** 10.1038/s41391-023-00681-3

**Published:** 2023-06-19

**Authors:** Ronald Tutrone, Ben Lowentritt, Brian Neuman, Michael J. Donovan, Elliot Hallmark, T. Jeffrey Cole, Yiyuan Yao, Claire Biesecker, Sonia Kumar, Vinita Verma, Grannum R. Sant, Jason Alter, Johan Skog

**Affiliations:** 1 Chesapeake Urology Research Associates, Baltimore, MD USA; 2 Icahn School of Medicine at Mt. Sinai, New York City, NY USA; 3 Exosome Diagnostics, a Bio-Techne Brand, Waltham, MA USA; 4 Department of Urology, Tufts University, Medford, MA USA

**Keywords:** Cancer, Outcomes research

Correction to: *Prostate Cancer and Prostatic Diseases* 10.1038/s41391-023-00675-1, published online 16 May 2023

The final sentence in the Results section of the abstract should read:

At 2.5 years, patients with low-risk EPI scores from both arms had less HGPC than high-risk EPI score patients (7.9% vs 27.3%, *p* < 0.001) and the EPI arm found 21.8% more HGPC than the SOC arm.

Instead, the final sentence in the Results section of the abstract currently reads:

At 2.5 years, patients with low-risk EPI scores from both arms had less HGPC than high-risk EPI score patients (7.9% vs 26.8%, *p* < 0.001) and the EPI arm found 18% more HGPC than the SOC arm.

The original article has been corrected.

