# Supplementary material for: ExoDx prostate test as a predictor of outcomes of high-grade prostate cancer – an interim analysis
Source: Prostate Cancer Prostatic Dis. 2023 May 16;26(3):596–601. doi: 10.1038/s41391-023-00675-1 (PMC10449627; doi:10.1038/s41391-023-00675-1)
Supplement: Supplementary file 1 — Supplemental Material [file 41391_2023_675_MOESM1_ESM.docx]

**ExoDx Prostate Test as a Predictor of Outcomes of High-Grade Prostate Cancer –**

**An Interim Analysis**

Ronald Tutrone, MD, Ben Lowentritt, MD, Brian Neuman, MD, Michael J. Donovan, PhD, MD, Elliot Hallmark, PhD, T. Jeffrey Cole, PhD, Yiyuan Yao, MS, Claire Biesecker, BA, Sonia Kumar, PhD, Vinita Verma, MPH, Grannum R Sant, MD, Jason Alter, PhD, Johan Skog, PhD

**Supplemental Material**

**Supplemental Table 1.** **EPI Scores and Gleason Grades stratified by study arm**

|  | **Arms Combined** | | **Arms Combined total** | **EPI Arm** | | **EPI Arm Total** | **SOC Arm** | | **SOC Arm Total** |
| --- | --- | --- | --- | --- | --- | --- | --- | --- | --- |
|  | **EPI≥15.6 (n=656)** | **EPI<15.6 (n=177)** | **(n=833)** | **EPI≥15.6 (n=328)** | **EPI<15.6 (n=83)** | **(n=411)** | **EPI≥15.6 (n=328)** | **EPI<15.6 (n=94)** | **(n=422)** |
| **Deferred** | 204 (31.1) | 84  (47.5) | 288  (34.6) | 69  (21.0) | 46  (55.4) | 115  (28.0) | 135  (41.2) | 38  (40.4) | 173  (41.0) |
|  |  |  |  |  |  |  |  |  |  |
| **Benign** | 179 (27.3) | 69  (39.0) | 248  (29.8) | 98  (29.9) | 30  (36.1) | 128  (31.1) | 81  (24.7) | 39  (41.5) | 120  (28.4) |
|  |  |  |  |  |  |  |  |  |  |
| **GG 1** | 94  (14.3) | 10  (5.6) | 104  (12.5) | 59  (18.0) | 3  (3.6) | 62  (15.1) | 35  (10.7) | 7  (7.4) | 42  (10.0) |
|  |  |  |  |  |  |  |  |  |  |
| **GG 2** | 90  (13.7) | 8  (4.5) | 98  (11.8) | 54  (16.5) | 3  (3.6) | 57  (13.9) | 36  (11.0) | 5  (5.3) | 41  (9.7) |
|  |  |  |  |  |  |  |  |  |  |
| **GG 3** | 56  (8.5) | 5  (2.8) | 61  (7.3) | 30  (9.1) | 1  (1.2) | 31  (7.5) | 26  (7.9) | 4  (4.3) | 30  (7.1) |
|  |  |  |  |  |  |  |  |  |  |
| **GG 4** | 15  (2.3) | 0  (0.0) | 15  (1.8) | 9  (2.7) | 0  (0.0) | 9  (2.2) | 6  (1.8) | 0  (0.0) | 6  (1.4) |
|  |  |  |  |  |  |  |  |  |  |
| **GG 5** | 18  (2.7) | 1  (0.6) | 19  (2.3) | 9  (2.7) | 0  (0.0) | 9  (2.2) | 9  (2.7) | 1  (1.1) | 10  (2.4) |
|  |  |  |  |  |  |  |  |  |  |
| **GG ≥2** | 179 (27.3) | 14  (7.9) | 193  (23.2) | 102  (31.1) | 4  (4.8) | 106  (25.8) | 77  (23.5) | 10  (10.6) | 87  (20.6) |
|  |  |  |  |  |  |  |  |  |  |

Data presented as N (%); Abbreviations: EPI=ExoDx Prostate (IntelliScore); GG=Grade Grade; SOC=Standard of Care
